# One Health—One Biofilm

**DOI:** 10.1186/s13567-022-01067-4

**Published:** 2022-07-07

**Authors:** Mario Jacques, François Malouin

**Affiliations:** 1 Regroupement de Recherche pour un Lait de Qualité Optimale (Op+lait), Saint-Hyacinthe, Québec J2S 2M2 Canada; 2 Faculté de Médecine Vétérinaire, Groupe de Recherche sur les Maladies Infectieuses en Production Animale (GREMIP), Université de Montréal, Saint-Hyacinthe, Québec J2S 2M2 Canada; 3 Département de Biologie, Faculté des Sciences, Université de Sherbrooke, Sherbrooke, Québec J1K 2R1 Canada

**Keywords:** Biofilms, bacterial aggregates, One Health

## Abstract

Bacterial biofilms are structured clusters of bacterial cells enclosed in a self-produced polymer matrix that are attached to a biotic or abiotic surface. This structure protects bacteria from hostile environmental conditions. There are also accumulating reports about bacterial aggregates associated but not directly adherent to surfaces. Interestingly, these bacterial aggregates exhibit many of the same phenotypes as surface-attached biofilms. Surface-attached biofilms as well as non-attached aggregates are ubiquitous and found in a wide variety of natural and clinical settings. This strongly suggests that biofilm/aggregate formation is important at some steps in the bacterial lifecycle. Biofilm/aggregate formation might therefore be important for some bacterial species for persistence within their host or their environment, while for other bacterial species it might be more important for persistence in the environment between infection of different individuals or even between infection of different hosts (humans or animals). This is strikingly similar to the One Health concept which recognizes that the health and well-being of humans, animals and the environment are intricately linked. We would like to propose that within this One Health concept, the One Biofilm concept also exists, where biofilm/aggregate formation in humans, animals and the environment are also intricately linked. Biofilm/aggregates could represent the unifying factor underneath the One Health concept. The One Biofilm concept would support that biofilm/aggregate formation might be important for persistence during infection but might as well be even more important for persistence in the environment and for transmission between different individuals/different hosts.

## 1. Bacterial biofilms

Bacterial biofilms are structured clusters of bacterial cells enclosed in a self-produced polymer matrix, attached to a biotic or abiotic surface [1]. This structure protects bacteria from hostile environmental conditions and guarantees the survival and spread of these communities. Bacteria in biofilms are more resistant/tolerant to antibiotics and disinfectants than planktonic cells and can withstand attacks from the host immune system [1, 2]. Although in vitro studies have focused mainly on single-species biofilms, multispecies biofilms are predominant in the context of host colonization and environmental conditions [3–5].

Intensive efforts are directed towards the development of new anti-biofilm molecules or strategies. The most frequent approaches that are currently investigated are blocking bacterial adhesion, killing persister cells, interfering with bacterial communication, inhibiting bacterial cooperation, degrading the polymeric matrix and stimulating dispersal of biofilms [6–9].

Cai [10] recently proposed that, in addition to the two main lifestyles (i.e., planktonic individuals and surface-attached biofilms), non-surface attached bacterial aggregates represent a third lifestyle. There are indeed accumulating reports in medical literature indicating that while some biofilms adhere to natural surfaces or artificial devices in the host, others may consist of bacterial aggregates that are not directly adherent to surfaces [11]. A characterization of biofilms associated with chronic infections in humans (e.g., lung infections, otitis media, osteomyelitis) revealed the presence of microbial cells aggregates ranging from  ~5 to 200 µm in diameter [12]. These bacterial aggregates, interestingly, share many of the same characteristics as surface-attached biofilms, such as increased antibiotic resistance/tolerance [13]. Aggregates were also observed in animals such as in the lungs of pigs infected with *Actinobacillus pleuropneumoniae* [14] and the mammary gland of cows infected with *Staphylococcus aureus* (I. Doghri, S. Dufour and M. Jacques, unpublished data). Furthermore, bacterial aggregates and biofilms are not mutually exclusive. *Salmonella* forms aggregates in conditions of biofilm formation, separate from the population that sticks to the solid surface at the air–liquid interface [15].

Surface-attached biofilms as well as non-attached aggregates are ubiquitous and found in a wide variety of natural environments (e.g., marine, freshwater and wastewater) and clinical situations (human and animal infections). Over the years, studies of bacterial genomes have documented the presence of a plethora of genes (sometimes even showing redundancy) involved in biofilm formation (e.g., for biosynthesis of polymeric matrix components [16]) as well as for biofilm regulation and modulation (e.g., quorum sensing, stringent response, and c-di-GMP signaling [17]). The fact that the huge and energy-consuming machinery necessary for matrix synthesis is present in many, if not all, genomes, and that these genes were conserved through evolution, strongly suggest that biofilm/aggregate formation is critical at certain steps in the bacterial lifecycle. For example, staphylococci produce one dominant extracellular polysaccharide (EPS) named polysaccharide intercellular adhesin (PIA) which production is mediated by the *ica* locus [18]. PIA and the *ica* locus were first described in *Staphylococcus epidermidis* but then also found in *Staphylococcus aureus* and other staphylococcal species with significant conservation [18]. Another illustration is the extracellular matrix of drar-positive cells in *Salmonella* that is comprised of protein polymers (curli fimbriae) and EPS (cellulose and an O-antigen capsule) [15]. The genes coding for these polymers are conserved throughout *Salmonella* and *Escherichia coli* [15].

Biofilm/aggregate formation, on the other hand, does not necessarily represent a virulence factor, but could rather be seen as a valuable asset for pathogenic bacteria’s persitence within a host during infection or between hosts. Moreover, the ability to produce large amounts of biofilms does not necessarily correlates with virulence. For example, our group has observed that non-virulent strains of *Glaesserella* (*Haemophilus*) *parasuis*, a porcine pathogen, have the ability to form robust biofilms in vitro in contrast to virulent, systemic strains [19]. Biofilm formation might therefore allow the non-virulent *G. parasuis* strains to colonize and persist in the upper respiratory tract of pigs. Conversely, the planktonic state of the virulent strains might allow them to disseminate within the host [19]. Hence, biofilm/aggregate formation might therefore be vital for persistence of some bacterial species within their host or environment, while it may have a more significant role in the ability of others to persist between infections of different individuals or hosts (humans or animals).

## 2. Similarities with the One Health concept

**Figure 1:**
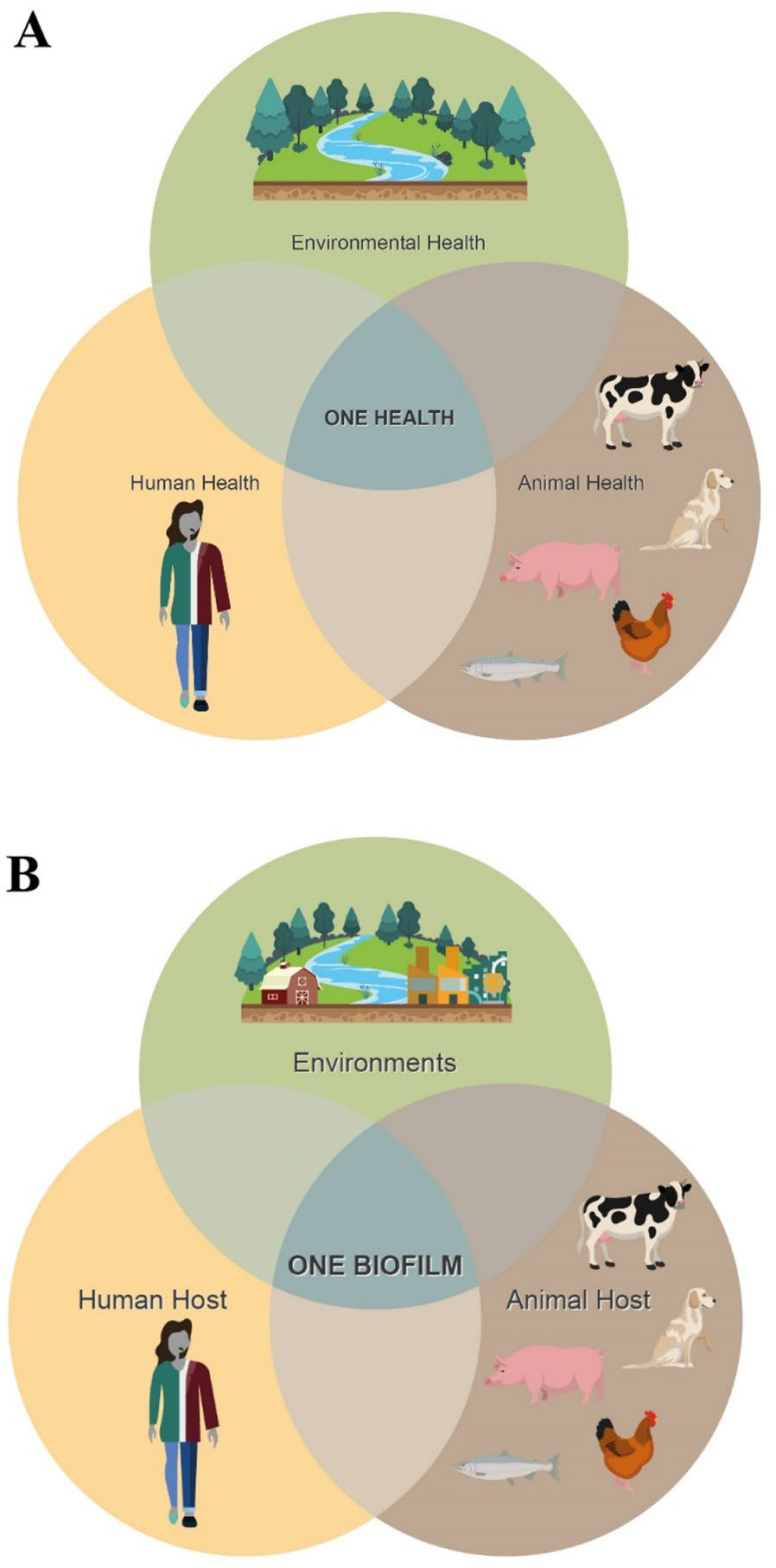
**The One Health and One Biofilm concepts. A** The One Health triad shows that the health of people, animals, and the environment are intricately linked. **B** Similarly, the One Biofilm triad proposes that bacterial biofilm/aggregate formation in humans, animals and the environments (e.g., natural environments but also farms, slaughterhouse or food-processing plants, and hospital settings) are also intricately linked.

Although several definitions exist for the One Health concept, the main point is awareness that the health and well-being of humans, animals (domestic and wild), and the environment are inextricably linked (Figure 1A) [20]. One Health also represents a collaborative, multisectoral, and transdisciplinary approach to address health threats at the human-animal-environment interface [20, 21]. The success of the One Health approach requires breaking down the barriers that still often separate human and veterinary medicine from ecological, evolutionary and environmental sciences [22] as well as socioeconomic and social policies. The One Health approach has been proven to be effective to tackle complex public health problems such as zoonotic diseases, antimicrobial resistance, food safety and food security, and other health threats shared by people, animals, and the environment such as pollution and climate changes [20]. For example, zoonoses (caused by pathogens such as *Salmonella*, *Campylobacter*, *Listeria*, and *E. coli*) are commonly spread at the human-animal-environment interface, where people and animals interact with each other in their shared environment [21, 23]. Zoonotic diseases can be foodborne, waterborne, vector-borne, or transmitted through direct contact with animals, indirectly by fomites or environmental contamination.

Similarly, biofilm/aggregate formation in humans, animals (domestic and wild), and the environment are probably also intricately linked, and we would like to propose the One Biofilm concept (Figure 1B). As such, the One Biofilm concept could be the cornerstone underlying the One Health concept. Biofilm formation is widespread among the microbial world and biosynthesis genes for biofilm production are present in most bacterial genomes. Biofilms can easily be evidenced, for multiple bacterial species, in the laboratory using in vitro assays (e.g., microtiter plates and flowcells). As indicated before, those facts strongly suggest that biofilm/aggregate formation is crucial at some steps in the bacterial lifecycle. The One Biofilm concept therefore supposes that biofilm/aggregate formation might be important for persistence during infection but might as well be even more important for persistence outside the host, in diverse environments (e.g., water, soil, surfaces at the farm, slaughterhouse or food-processing plants, hospital settings) and for transmission between different individuals and/or different hosts. We may not always understand (or have not discovered yet) where the biofilm is most important (i.e., which lifecycle stages). The One Biofilm concept is adding another layer of complexity to the One Health concept. A collaborative, multi-stakeholder, multisectoral, and transdisciplinary approach is therefore preferred to address complex problems involving biofilms or bacterial aggregates including the development of new and effective strategies to prevent or control their formation and to prevent persistence and spread of microbial pathogens across environments, animals and people. The One Biofilm concept is a proposition and we would invite researchers to build on the present proposition and to add observations to further strengthen and refine this new concept.
